# Adaptation of Fibril-Reinforced Poroviscoelastic Properties in Rabbit Collateral Ligaments 8 Weeks After Anterior Cruciate Ligament Transection

**DOI:** 10.1007/s10439-022-03081-1

**Published:** 2022-09-21

**Authors:** Gustavo A. Orozco, Aapo Ristaniemi, Mehrnoush Haghighatnejad, Ali Mohammadi, Mikko A. J. Finnilä, Simo Saarakkala, Walter Herzog, Hanna Isaksson, Rami K. Korhonen

**Affiliations:** 1 Department of Applied Physics, University of Eastern Finland, Yliopistonranta 1, 70210 Kuopio, Finland; 2 Department of Biomedical Engineering, Lund University, Box 188, 221 00 Lund, Sweden; 3 AO Research Institute Davos, Davos, Switzerland; 4 Research Unit of Medical Imaging, Physics and Technology, University of Oulu, Oulu, Finland; 5 Department of Diagnostic Radiology, Oulu University Hospital, Oulu, Finland; 6 Human Performance Laboratory, Faculty of Kinesiology, University of Calgary, Calgary, Canada

**Keywords:** Medial/lateral collateral ligament, Anterior cruciate ligament transection, Finite element model, Tissue adaptation, Rabbit model

## Abstract

Ligaments of the knee provide stability and prevent excessive motions of the joint. Rupture of the anterior cruciate ligament (ACL), a common sports injury, results in an altered loading environment for other tissues in the joint, likely leading to their mechanical adaptation. In the collateral ligaments, the patterns and mechanisms of biomechanical adaptation following ACL transection (ACLT) remain unknown. We aimed to characterize the adaptation of elastic and viscoelastic properties of the lateral and medial collateral ligaments eight weeks after ACLT. Unilateral ACLT was performed in six rabbits, and collateral ligaments were harvested from transected and contralateral knee joints after eight weeks, and from an intact control group (eight knees from four animals). The cross-sectional areas were measured with micro-computed tomography. Stepwise tensile stress-relaxation testing was conducted up to 6% final strain, and the elastic and viscoelastic properties were characterized with a fibril-reinforced poroviscoelastic material model. We found that the cross-sectional area of the collateral ligaments in the ACL transected knees increased, the nonlinear elastic collagen network modulus of the LCL decreased, and the amount of fast relaxation in the MCL decreased. Our results indicate that rupture of the ACL leads to an early adaptation of the elastic and viscoelastic properties of the collagen fibrillar network in the collateral ligaments. These adaptations may be important to consider when evaluating whole knee joint mechanics after ACL rupture, and the results aid in understanding the consequences of ACL rupture on other tissues.

## Introduction

Knee joint ligaments play an essential role during daily activities providing mechanical stability and preventing excessive motion. Knee ligaments are flexible and strong fibrous tissues, which allow movement between the femur and tibia offering suitable resistance to applied forces.^8^ However, traumatic injuries, such as rupture of the anterior cruciate ligament (ACL), alter knee function and result in mechanical adaptation of the remaining knee joint structures, such as the adjacent ligaments, bone, infrapatellar fat pad, menisci, and articular cartilage.^4,61^ In several animal studies, the effect of ACL transection (ACLT) on bone, menisci, and cartilage has been studied with the goal to comprehend the patterns and mechanisms of biomechanically-driven tissue adaptation.^4,6,10,13,25,27,31^ Nevertheless, only few studies have reported the physiological and biomechanical alterations of the collateral ligaments within the knee joint following ACLT.^28,35,37,54^

Experimental and numerical studies have investigated collateral ligament mechanics following ACLT. For instance, large stresses were measured on the collateral ligaments of human cadaveric knees under weight-bearing conditions after ACLT.^16,17^ Likewise, the medial collateral ligament (MCL) has been shown to experience a significant increase in forces under anterior tibial loading in ACL-transected cadaveric knees.^47^ Computational models also predict excessive stresses on MCL under different loading scenarios in ACL-deficient knees.^42,50^ Studies combining ACL and MCL injuries have reported excessive loading as a plausible mechanism for reducing the MCL healing compared to the isolated MCL injury model.^1,5^ Regarding the lateral collateral ligament (LCL), large strain concentrations were observed in LCL under axial loading on the tibia following ACL rupture.^26^ In contrast, no significant changes have been reported in LCL tensile mechanical properties following ACLT.^56^ However, significant shortening of the LCL fiber bundles was described after ACL injury in a clinical in vivo imaging study.^57^

The main components of ligaments are water, collagen type I, elastin, proteoglycans, and cells.^62^ Structurally, the collagen network controls the tensile response,^53^ proteoglycans attract water and affect collagen fibril separation,^19,65^ and elastin provides load support under deformation transverse to the primary collagen axis and in shear.^15^ Biomechanically, ligaments exhibit a complex viscoelastic behavior resulting from the interactions among their constituents, particularly the fibrillar collagen network that controls the tensile behavior.^59^ The stress-relaxation of ligaments in tension has been characterized by a two-relaxation-time response,^12,48,49^ i.e., a double-exponential decay of the stress as a function of time, with a distinct fast and long-term relaxation rates of the collagen network. Moreover, stress-relaxation of ligaments increases with increasing strains,^12,23,29,48,49^ i.e., higher difference between the peak and equilibrium stress when the strain is higher. Numerical models have been developed to study the poroviscoelasticity of soft tissues.^51,52^ Recently, Ristaniemi *et al.* developed a fibril-reinforced poroviscoelastic material model capable of capturing the complex relaxation phenomena in bovine ACLs.^45^ Subsequently, this model was applied to characterize the mechanical properties of other knee joint ligaments.^43^ Importantly, this model allows for the determination of alterations in the fibril-reinforced properties of ligaments following ACLT and the understanding of the potential mechanisms that underly the adaptative processes.

In the present study, we aimed to characterize the biomechanical adaptation of the collateral ligaments eight weeks after ACLT using a fibril-reinforced poroviscoelastic material model. We hypothesize that the cross-sectional area and the viscoelastic properties of the collagen network change substantially following ACL transection due to changes in knee joint loading. The material properties of collateral ligaments were compared with histopathological osteoarthritis severity in the cartilage. The results of our investigation contribute to understanding the adaptive mechanisms of collateral ligaments after traumatic injuries as well as may help models of the whole joint to improve understanding of mechanisms leading to post-traumatic OA following alterations in the joint loading environment. In addition, this study provides insights into changes in tissues that are not directly known to be damaged.

## Materials and Methods

### Animal Model and Sample Preparation

**Figure 1:**
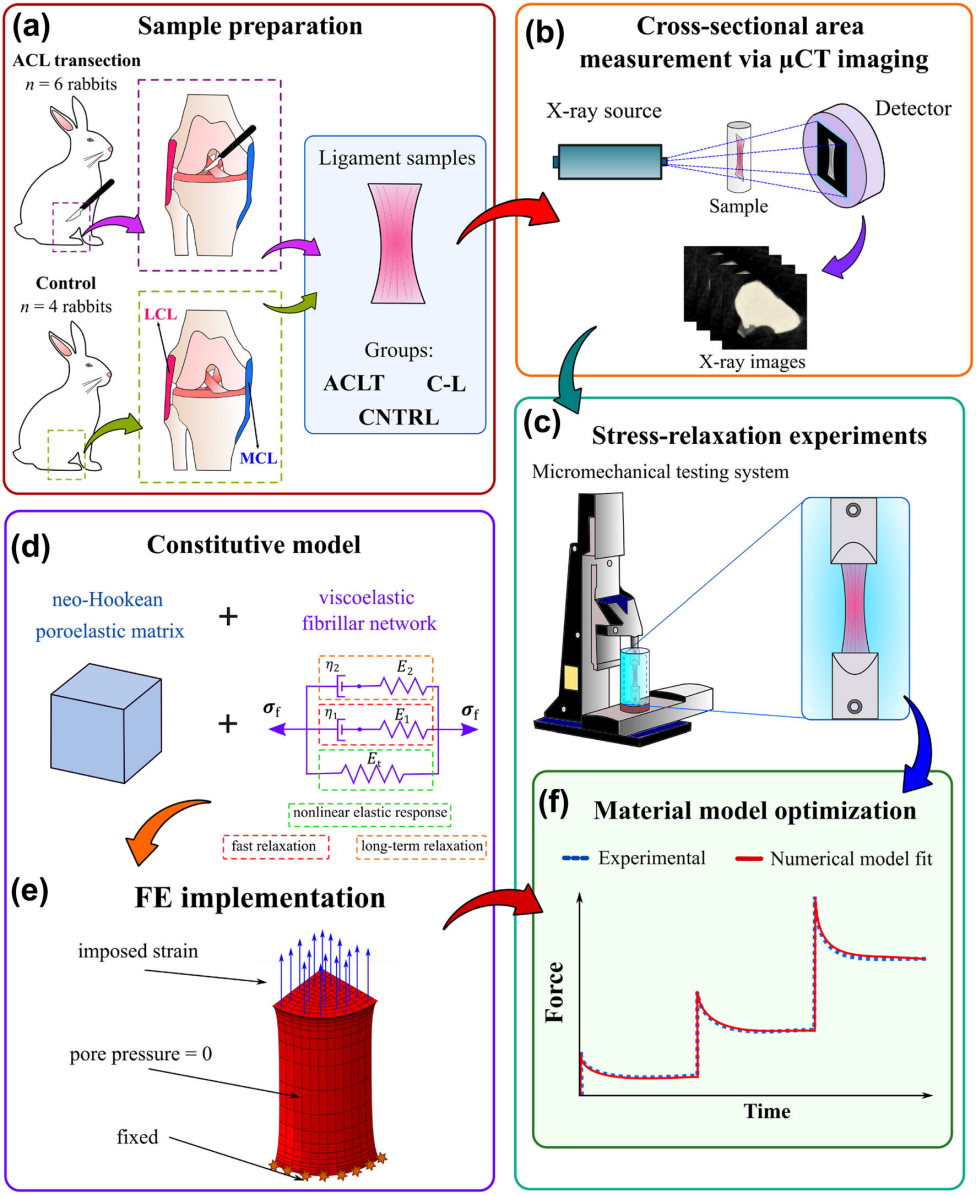
Workflow of the study. (a) Medial (MCL) and lateral collateral ligaments (LCL) were carefully dissected from Anterior Cruciate Ligament-transected (ACLT), contralateral (C-L), and control (CNTRL) rabbit knee joints. **b** The cross-sectional area was measured using micro-computed tomography (µCT) imaging data. (c) Tensile stress-relaxation experiments of the collateral ligaments were conducted using a micromechanical testing system. (d) A fibril-reinforced poroviscoelastic material model^43^ was implemented in a finite element model to replicate each stress-relaxation experiment. (f) The force–time output of the numerical model was fit to the experimental force–time curve measured at the toe region of the stress–strain curve to determine the material properties of each sample.

A unilateral ACLT procedure was done randomly on the left or right knee joint in six skeletally mature female New Zealand white rabbits (Oryctolagus cuniculus, age 12 months at the time of surgery, weight 4.8 ± 0.1 kg). The animals recovered postoperatively on a heating pad covered with a blanket until they were mobile and returned to their cages, where they were allowed to move freely. All rabbits showed normal activity, without indication of altered behavior after surgery. Eight weeks post-surgery, the rabbits were euthanized under controlled anesthesia.^34^ Both ACL-transected and intact contralateral (C-L) knee joints were collected. In addition, knee joints from healthy rabbits (CNTRL, *n* = 8 knees, from four rabbits, weight 4.57 ± 0.35 kg) were utilized as a control group (Fig. 1a). All procedures were carried out according to the guidelines of the Canadian Council on Animal Care and were approved by the committee on Animal Ethics at the University of Calgary. Sample size estimation was conducted before animal experiments as part of previous studies^18,20^ (see details in Supplementary material in Huang *et al.*^18^).

Medial collateral ligaments (MCL) and lateral collateral ligaments (LCL) were carefully dissected from ACLT, C-L, and CNTRL rabbit knee joints. The MCL and LCL samples were immersed in isotonic phosphate-buffered saline (PBS) solution and stored at − 20 °C immediately after dissection. Prior to measurements, samples were thawed at room temperature. For the cross-sectional area measurements, surplus PBS was dried from the sample surface, and excess adipose tissue was carefully removed. Subsequently, the mechanical measurement was conducted in a PBS solution (temperature approx. 21 °C) with enzyme inhibitors benzamidine hydrochlorid hydrate (0.78 g l − 1, Sigma-Aldrich Co., St Louis, MO, USA) and ethylenediaminetetraacetic acid disodium salt (1.86 g l − 1, EDTA VWR International, Radnor, PA, USA).

### Cross-Sectional Area Measurement

Samples were placed in plastic vials for micro-computed tomography (µCT) imaging (Skyscan 1172; Bruker microCT). The voltage of X-ray tube for the µCT was set to 60 kV. The images were reconstructed in NRecon software with a voxel size of 34.72 × 34.72 × 34.72 µm^3^. DICOM image dataset with a size of 328 × 328 pixels x sample height was loaded in Image-J software (Rasband, W.S., ImageJ, U. S. National Institutes of Health, Bethesda, Maryland, USA, https://imagej.nih.gov/ij/) and three sections (middle image ± 0.1 mm) were selected for the measurement of cross-sectional area. The cross-section was manually segmented in each image and the area was computed in Image-J. For each ligament sample, the final area was the average from the three images (Fig. 1b).

### Stress-Relaxation Testing

Tensile stress-relaxation testing was conducted using a micromechanical testing system (Mach-1 v500csst, Biomomentum Inc., Laval, QC, Canada), with a uniaxial 250 N load cell (resolution 12.5 mN) and custom jaw-type clamps (Fig. 1c). The clamps were put into contact and the vertical displacement was set to zero, after which the vertical stage reading (accuracy 0.1 µm) showed the clamp-to-clamp distance. The sample was secured between the clamps by tightening the clamping screw to a moment of 2 Nm which ensured appropriate and constant clamping for all samples. The clamp surfaces were covered with sandpaper (Mirox P120, Mirka Oy, Uusikaarlepyy, Finland) to enhance friction at the clamp-tissue interface. Tensile stress of 0.05 MPa^14,44^ was utilized to eliminate slack and to establish the zero-load length (measured as the clamp-to-clamp length). Preconditioning was performed with 10 loading–unloading cycles to 6% strain with a strain rate of 2%/s, followed by re-determination of the zero-load length. The 6% strain did not induce any damage to the samples since the equilibrium stress–strain curves were linear (below yield point) and unexpected changes in the force of the stress-relaxation were not observed. This preconditioning protocol was repeated five times to ensure a stable mechanical response.^9^ The sample was subsequently allowed to recover for 10 min, after which the zero-load length was verified, and the mechanical measurement was started. An incremental stress-relaxation experiment was performed using four consecutive steps of 2% strain (*i.e.*, relaxation at strain levels of 2, 4, 6, and 8%), with 10%/s ramp velocity and a 30-min relaxation period at each increment (Fig. 1c). The first three consecutive steps (from 0 to 6%) were used to obtain the fibril-reinforced poroviscoelastic material properties while the last step (6% to 8%) was used to calculate the stiffness of the collateral ligaments.

### Finite Element Model

A finite element (FE) model of each collateral ligament was constructed to replicate the stress-relaxation experiments. Based on a previous study, one baseline FE model was used with 2592 C3D8P elements in ABAQUS (v. 2021, Dassault Systèmes, Johnston, RI, USA) and scaled according to individual sample geometries.^43^ A 3D quarter-cylinder geometry was created using both zero-load length and the cross-sectional area acquired from the experimental measurements. In order to reproduce the stress-relaxation test, the following boundary conditions were applied: bottom surface displacements were restricted, and top surfaces were assigned vertical displacements according to the experiment. Displacements at the symmetry planes were restricted in the perpendicular direction, and free fluid flow was allowed at the outer surface by assigning zero pore pressure (Fig. 1e).

### Fibril-Reinforced Poroviscoelastic Material Model

The knee collateral ligaments were modeled using a fibril-reinforced poroviscoelastic constitutive model. The model assumes that the tissue is composed of solid and fluid matrices. The solid matrix is divided into a porous non-fibrillar part and a viscoelastic fibrillar network. The total stress is given by

1 $${\varvec{\sigma}}_{{{\text{tot}}}} = {\varvec{\sigma}}_{{\text{s}}} + {\varvec{\sigma}}_{{{\text{fl}}}} = \user2{ \sigma }_{{\text{f}}} + {\varvec{\sigma}}_{{{\text{nf}}}} - p{\mathbf{I}}$$

where ${{\varvec{\sigma}}}_{\mathrm{tot}}$ is the total stress tensor, ${{\varvec{\sigma}}}_{\mathrm{s}}$ and ${{\varvec{\sigma}}}_{\mathrm{fl}}$ represent the stress tensors of the solid and fluid matrices, respectively, *p* is the hydrostatic pressure, $\mathbf{I}$ is the unit tensor, and ${{\varvec{\sigma}}}_{\mathrm{f}}$ and ${{\varvec{\sigma}}}_{\mathrm{nf}}$ are the stress tensors of the fibrillar and non-fibrillar matrices, respectively. A neo-Hookean material was used to define the non-fibrillar component, in which the stress tensor is given by

2 $${\varvec{\sigma}}_{{{\text{nf}}}} = \frac{1}{2}K_{{{\text{nf}}}} \left( {J - \frac{1}{J}} \right){\mathbf{I}} + \frac{{G_{{{\text{nf}}}} }}{J}\left( {{\mathbf{F}} \cdot {\mathbf{F}}^{{\text{T}}} - J^{\frac{2}{3}} { }{\mathbf{I}}} \right),$$

where ${K}_{\mathrm{nf}}$ and ${G}_{\mathrm{nf}}$ are the bulk and the shear moduli of the non-fibrillar matrix and $J$ is the determinant of the deformation gradient tensor **F**. The bulk (${K}_{\mathrm{nf}}$) and shear (${G}_{\mathrm{nf}}$) modulus were established as

3 $$K_{{{\text{nf}}}} = \frac{{E_{{{\text{nf}}}} }}{{3\left( {1 - 2\nu_{{{\text{nf}}}} } \right)}} ,$$

4 $$G_{{{\text{nf}}}} = \frac{{E_{{{\text{nf}}}} }}{{2\left( {1 + \nu_{{{\text{nf}}}} } \right)}} ,$$

where ${E}_{\mathrm{nf}}$ and ${\nu }_{\mathrm{nf}}$ are the Young’s modulus and the Poisson’s ratio of the non-fibrillar matrix. The fluid flow in the non-fibrillar matrix was simulated according to Darcy’s law:

5 $$q = - k\nabla p ,$$

where $q$ is the fluid flux in the non-fibrillar matrix, $\nabla p$ is the hydrostatic pressure gradient vector across the region and $k$ is the hydraulic permeability. The hydraulic permeability is defined to be strain-dependent:

6 $$k = k_{0} \left( {\frac{e + 1}{{1 + e_{0} }}} \right)^{M} = k_{0} J^{M} ,$$

where ${k}_{0}$ is the initial permeability, *M* is the permeability strain-dependency coefficient, and $e$ and ${e}_{0}$ are the current and initial void ratios. ${k}_{0}$ and *M* were fixed to 2.9 × 10^–15^ m^4^ N^−1^ s^−1^ and 7.9, respectively^60^ due to their negligible effect on the overall response in tension.^39,45^ The value for the initial void ratio was set to 3.5 (corresponding to the fluid fraction of 0.776) based on a previous experimental study.^46^ Additionally, the Poisson´s ratio of the non-fibrillar matrix was fixed at 0.48 based on Vergari *et al.*^58^

The viscoelastic fibrillar network was modeled by a nonlinear elastic spring in parallel with two Maxwell elements (Fig. 1d). It was assumed that collagen fibrils support tension only, *i.e.*, acting only at positive fibril strains (${\varepsilon }_{\mathrm{f}}$ > 0). The first three relaxation steps (from 0 to 6% strain) were used to characterize the fibril-reinforced poroviscoelastic material properties. The nonlinear spring (modulus ${E}_{\mathrm{t}}$) describes the nonlinear elastic behavior of the collagen network at the toe region (*i.e.*, the equilibrium stress increases nonlinearly as a function of strain).^62–64^ The two Maxwell elements correspond to the fast and long-term relaxations observed within the fibrillar network.^12,48,49^ The elastic parts in the Maxwell elements depend linearly on fibril strain (moduli ${E}_{1}$ and ${E}_{2}$), which describe the magnitude of fast relaxation (difference between the peak and equilibrium stress) and increase in the magnitude of long-term relaxation at high strain levels.^12,23,29,48,49^ Finally, damping components of the Maxwell elements describe the fast (${\eta }_{1}$) and long-term (${\eta }_{2}$) relaxation responses in the collagen network (Fig. 1d). A full description of the parameters of the material model can be found in Table 1.

**Table 1:** Description of the material model parameters used for ligaments.

Property	Symbol	Description
Elastic modulus of the non-fibrillar matrix	${E}_{\mathrm{nf}}$	Elastic behavior of the non-fibrillar matrix
Poisson’s ratio of the non-fibrillar matrix	${\nu }_{\mathrm{nf}}$	Poisson’s ratio of the non-fibrillar matrix
Initial permeability	${k}_{0}$	Initial permeability of the non-fibrillar matrix
Permeability strain-dependency coefficient	$M$	Coefficient describing permeability strain dependency
Initial void ratio	${e}_{0}$	Initial void ratio
Nonlinear fibrillar network modulus	${E}_{\mathrm{t}}$	Nonlinear elastic behavior of the fibrillar network
Fibrillar network modulus	${E}_{1}$	Elastic part of the Maxwell element describing the magnitude of fast relaxation or recruitment of viscoelasticity of the fibrillar network
Damping coefficient	${\eta }_{1}$	Damping component of the Maxwell element describing fast relaxation of the fibrillar network
Fibrillar network modulus	${E}_{2}$	Elastic part of the Maxwell element describing the magnitude of long-term relaxation or recruitment of viscoelasticity of the fibrillar network
Damping coefficient	${\eta }_{2}$	Damping component of the Maxwell element describing long-term relaxation of the fibrillar network

### Material Parameter Optimization

Material parameters of each sample (${{E}_{\mathrm{nf}},E}_{1},{E}_{2}{,E}_{\mathrm{t}},{\eta }_{1}$, and ${\eta }_{2}$) were determined by fitting the reaction force–time output of the FE model to the experimentally measured force–time curve from the first three consecutive steps (2% to 6% strain) (Fig. 1f). For the fitting process, the objective function $f=\sqrt{1-{R}^{2}}$ was minimized using *fminsearch*-function in MATLAB (R2018b, The MathWorks, Inc., Natick, MA, USA), as described before.^43^ In addition, the root mean square deviation (RMSD) was also calculated for the optimized result.

### Articular Cartilage Histopathology

The osteoarthritis severity of the articular cartilage of the rabbit knees was defined according to the Osteoarthritis Research Society International (OARSI) histopathological grading system.^24^ The histopathological evaluations were conducted in a previous investigation^18^ and adopted here to explore the potential relationship between cartilage degeneration and the fibril-reinforced poroviscoelastic material parameters of the collateral ligaments. Briefly, three 3-µm-thick sections were cut from the central load-bearing area of each location of the rabbit knee joints (*i.e.*, lateral tibia; medial tibia; lateral femur; medial femur; groove; patella), and the sections were stained with Safranin-O. Microscopic images of the stained sections were collected with a digital pathology slide scanner. The OARSI scores were assessed from these images according to the established histopathology standards described by the OARSI.^40^

### Statistical Analyses

Statistical comparisons of cross-sectional area and the material parameters (dependent variables) between the sample groups (control, contralateral and ACL-transected) were performed using a linear mixed model^32^ with the group as a fixed variable, and the animal as a random variable, followed by the least significant difference (LSD) post-hoc test. In addition, Spearman’s correlation analysis was conducted to evaluate potential relationships between the OARSI grades assessed for tibial, femoral, and patellar cartilages^18^ and the fibril-reinforced poroviscoelastic material parameters of the collateral ligaments. In all analyses, the level of statistical significance was set to $\alpha$= 0.05. The analyses were conducted using IBM SPSS Statistics 27.0.0.0 (SPSS Inc., IBM Company, Armonk, NY, USA), and were made separately for MCL and LCL.

## Results

### Cross-Sectional Area and Stiffness

**Figure 2:**
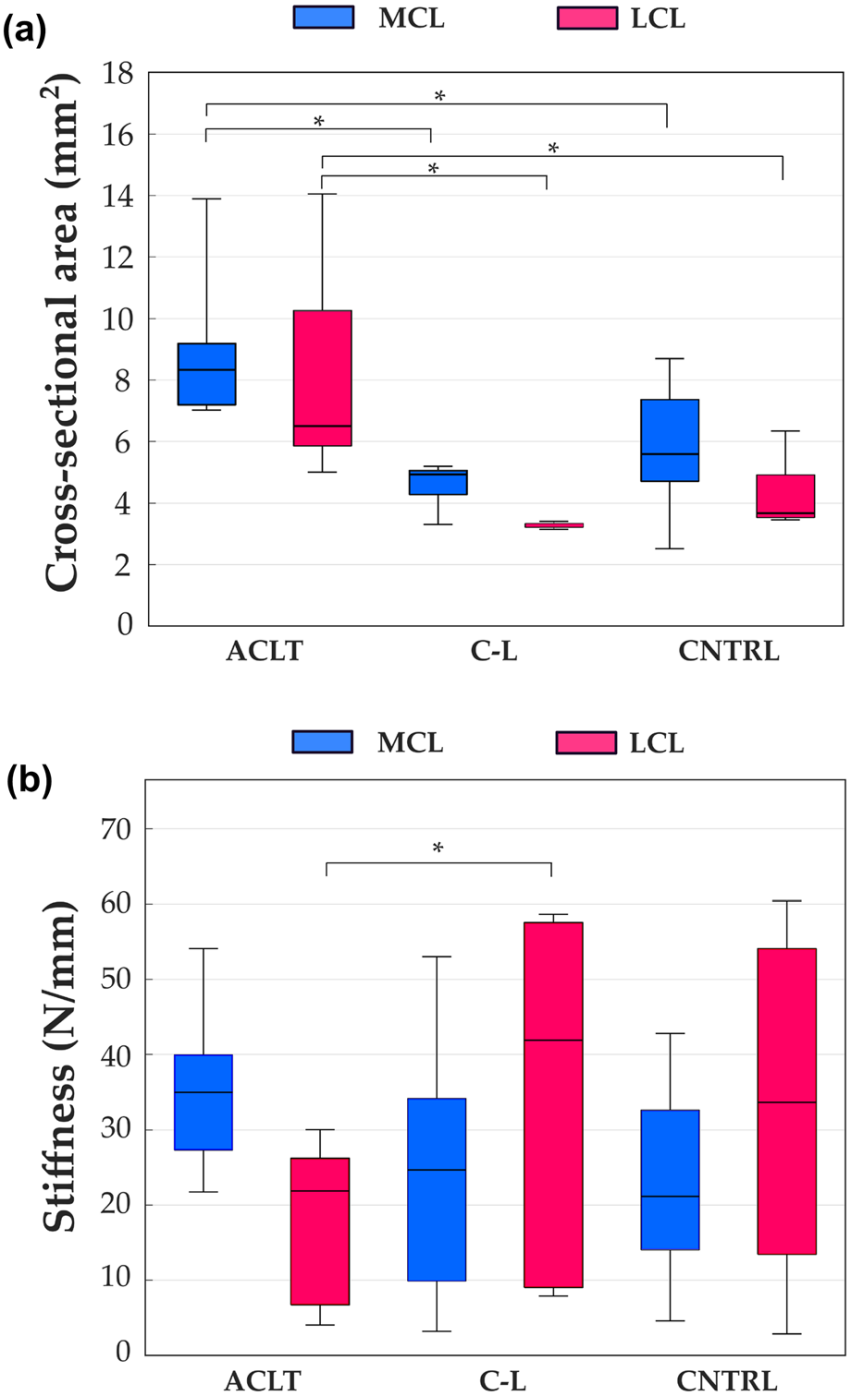
(a) The cross-sectional area and (b) the stiffness of the medial collateral ligament (MCL, blue) and the lateral collateral ligament (LCL, magenta) samples for the Anterior Cruciate Ligament-transected (ACLT), contralateral (C-L), and control (CNTRL) groups, respectively are shown. The boxplot shows median (horizontal line), 25th and 75th percentile (colored box), and minimum and maximum values (bars). *α < 0.05.

The cross-sectional area of the MCL was greater in the ACLT knees (8.99 mm^2^) compared with the contralateral (4.61 mm^2^, $p$=0.01) and the control group joints (5.81 mm^2^, $p$=0.018) (Fig. 2a, blue bars). Furthermore, the cross-sectional area of the LCL was greater in the ACLT (8.13 mm^2^) compared with the contralateral (3.26 mm^2^, $p$=0.02) and the control group knees (4.25 mm^2^, $p$=0.04) (Fig. 2a, magenta bars). In contrast, there were no statistical differences in the cross-sectional area of ligaments between contralateral and control group knees. The stiffness of the LCL was lower in the ACLT (17.66 N/mm) compared with the contralateral group (36.11 N/mm, $p$ =0.04) (Fig. 2b, magenta bars). There was no difference in MCL stiffness between any of the groups (Fig. 2b, blue bars).

### Fibril-Reinforced Poroviscoelastic Material Properties

**Figure 3:**
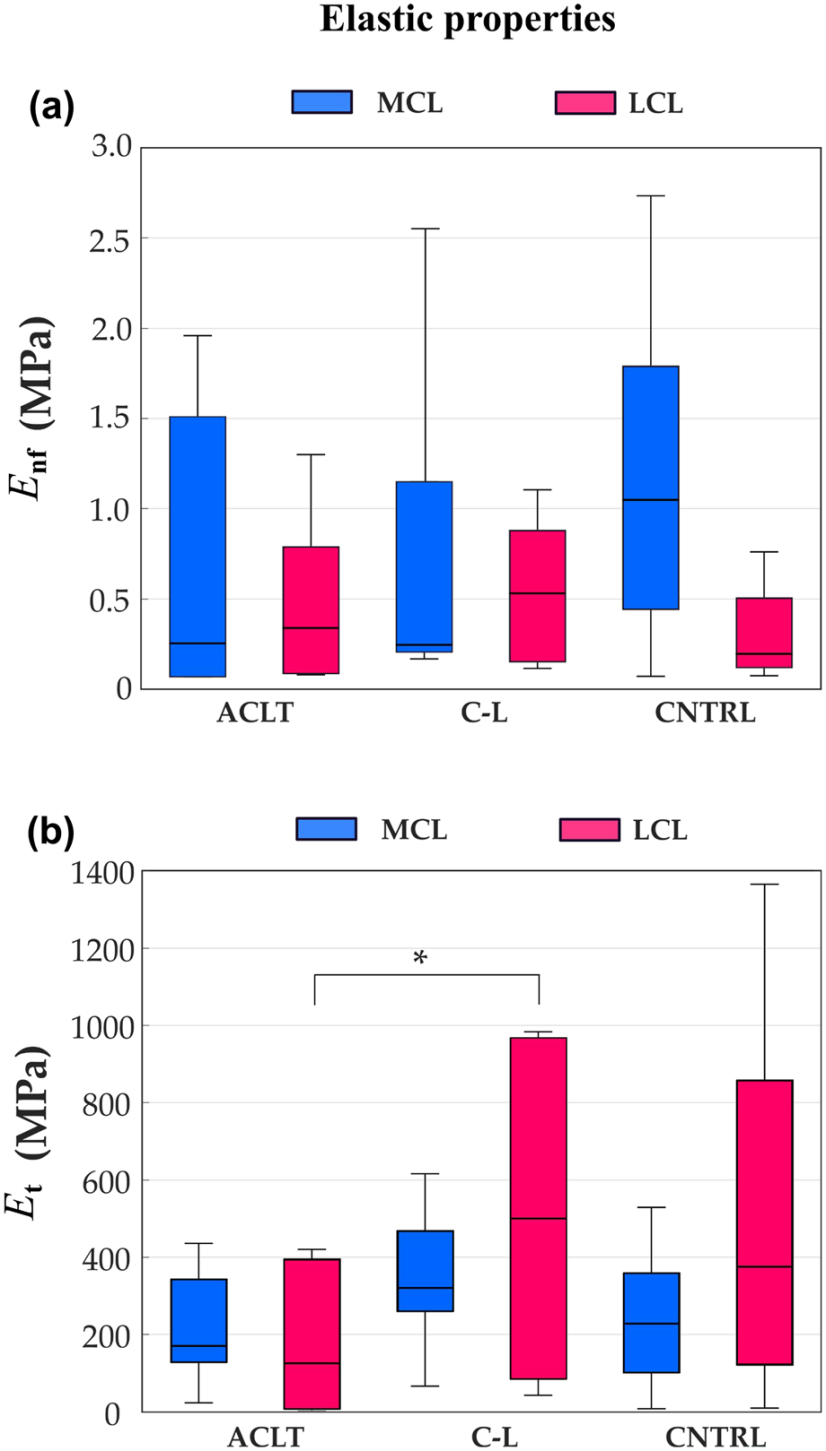
Elastic properties of the medial collateral ligament (MCL, blue) and the lateral collateral ligament (LCL, magenta) samples for the Anterior Cruciate Ligament-transected (ACLT), contralateral (C-L), and control (CNTRL) groups, respectively are shown. (a) Elastic modulus of the non-fibrillar matrix. (b) Nonlinear elastic fibrillar network modulus. The boxplot shows median (horizontal line), 25th and 75th percentile (colored box), and minimum and maximum values (bars). *α < 0.05.

The fibril-reinforced poroviscoelastic material model fitted the experimental results well (R^2^ mean value 0.98, range 0.92–1.00; RMSD mean value 5.6, range 1.4–9.1). The nonlinear elastic fibrillar network modulus ${E}_{\mathrm{t}}$ of the LCL was lower in the ACLT (189 MPa) compared with the contralateral group (513 MPa, $p$=0.04). The MCL fibrillar network modulus ${E}_{\mathrm{t}}$ showed no differences between any of the groups. In addition, no statistical differences in the elastic modulus of the non-fibrillar matrix, ${E}_{\mathrm{nf}}$ were observed between the groups (Fig. 3).

**Figure 4:**
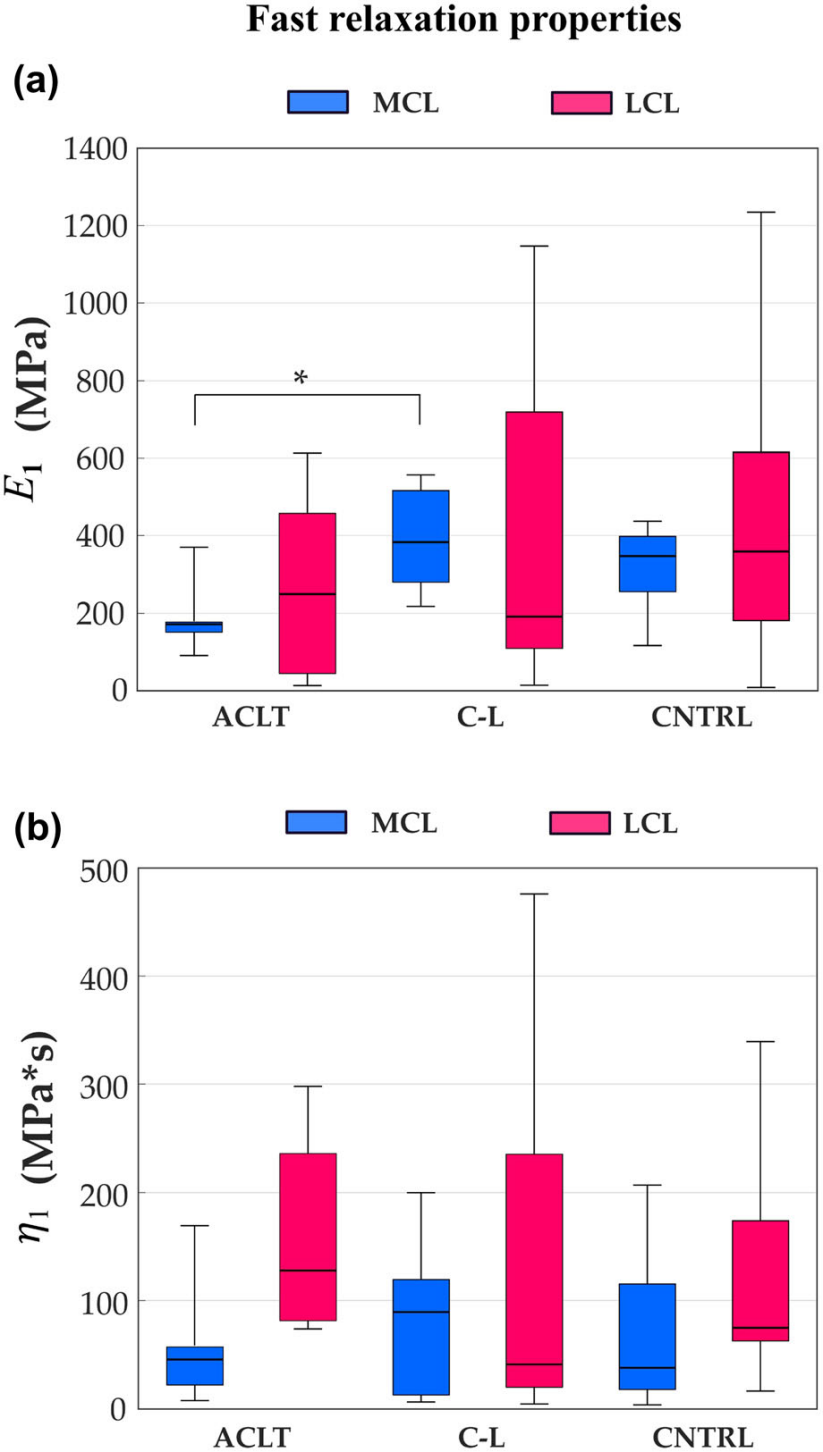
Fast relaxation properties of the medial collateral ligament (MCL, blue) and the lateral collateral ligament (LCL, magenta) samples for the Anterior Cruciate Ligament-transected (ACLT), contralateral (C-L), and control (CNTRL) groups, respectively are shown. (a) The elastic part of the Maxwell element describing the magnitude of fast relaxation or recruitment of viscoelasticity of the fibrillar network. (b) Damping component of the Maxwell element describing fast relaxation of the fibrillar network. The boxplot shows median (horizontal line), 25th and 75th percentile (colored box), and minimum and maximum values (bars). *α < 0.05.

**Figure 5:**
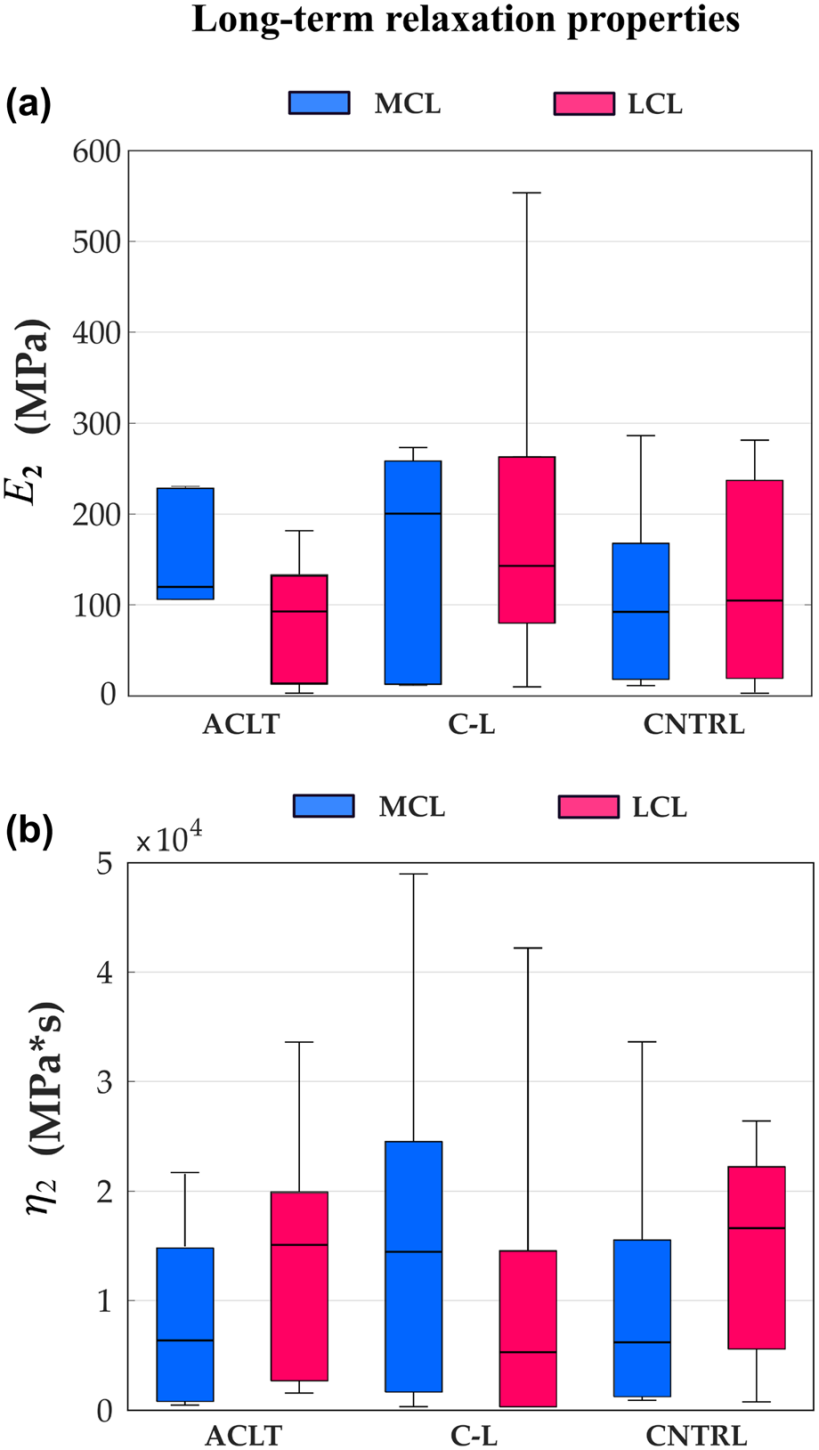
Long-term relaxation properties of the medial collateral ligament (MCL, blue) and the lateral collateral ligament (LCL, magenta) samples for the Anterior Cruciate Ligament transected (ACLT), contralateral (C-L), and control (CNTRL) groups, respectively are shown. (a) The elastic part of the Maxwell element describing the magnitude of long-term relaxation or recruitment of viscoelasticity of the fibrillar network. (b) Damping component of the Maxwell element describing long-term relaxation of the fibrillar network. The boxplot shows median (horizontal line), 25th and 75th percentile (colored box), and minimum and maximum values (bars). *α < 0.05.

The magnitude of fast relaxation (described by ${E}_{1}$) of the MCL was lower in the ACLT (190 MPa) compared with the contralateral group (389 MPa, $p$=0.008), but that of LCL showed no differences between groups (Fig. 4a). No statistical differences were found between the groups in the damping coefficient ${\eta }_{1}$ (Fig. 4b), in the magnitude of long-term relaxation (described by ${E}_{2}$), or in the damping coefficient ${\eta }_{2}$ (Fig. 5).

**Figure 6:**
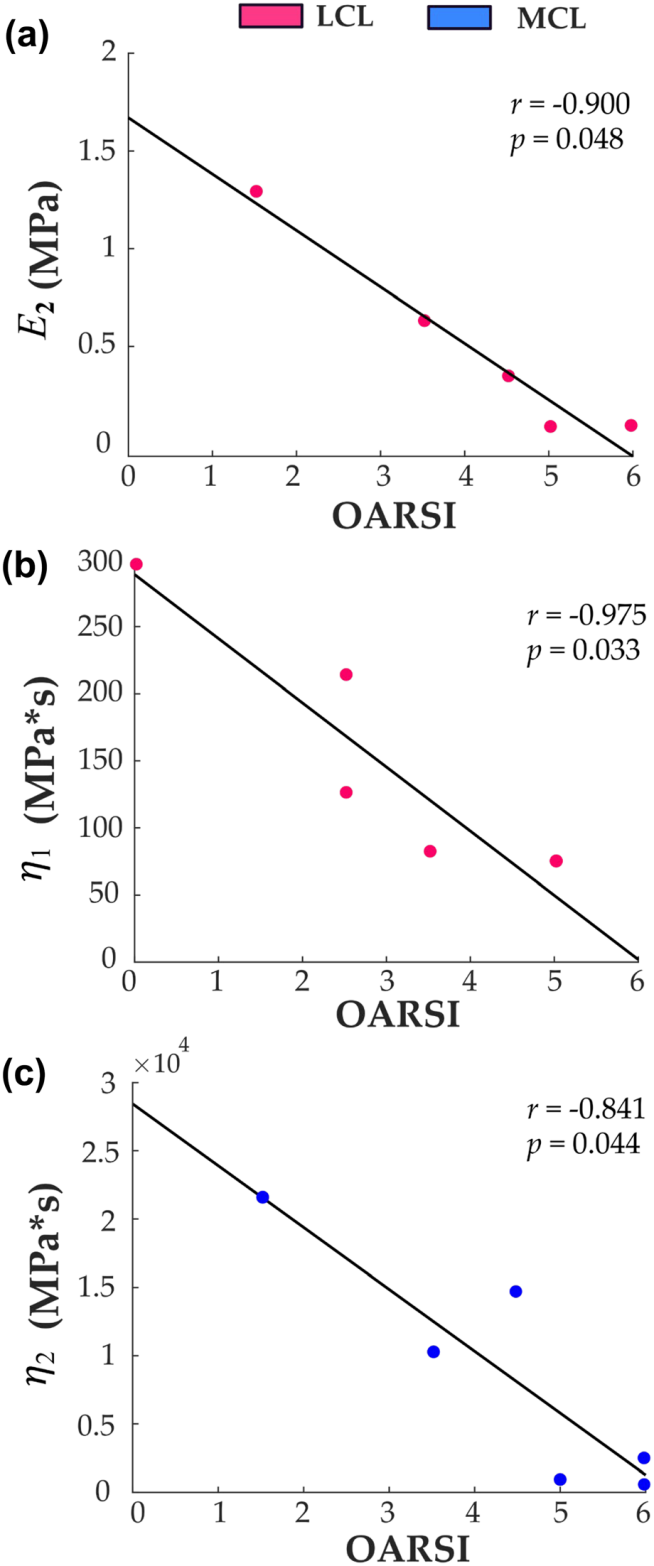
Scatter plots between OARSI grades and fibril-reinforced poroviscoelastic properties of collateral ligaments. Statistically significant (Spearman’s) correlations are presented from ACLT group and the OARSI scores. (a) lateral femur and the elastic modulus of the non-fibrillar matrix, (b) patella and the damping component describing fast relaxation of the fibrillar network, and (c) lateral femur and damping component describing long-term relaxation of the fibrillar network.

Significant correlations were observed between certain fibril-reinforced poroviscoelastic material parameters and OARSI grades in the ACLT group (Table 2). Specifically, the non-fibrillar matrix modulus, ${E}_{\mathrm{nf}},$ and the damping coefficient, ${\eta }_{1},$ of the LCL was negatively correlated with the OARSI scores of the lateral femoral cartilage (*p* = 0.048, *r* =  −0.90) and patella (*p* = 0.033, *r* =  −0.975). For MCL, only the damping coefficient, ${\eta }_{2},$ was negatively correlated with the OARSI scores of the lateral femoral cartilage (*p* = 0.044, *r* =  −0.84) (Fig. 6). There were no significant correlations between the material properties of the collateral ligament and any of the OARSI scores in the contralateral and control groups (Tables 3 and 4, respectively).

**Table 2:** Spearman’s correlations between optimized fibril-reinforced poroviscoelastic material parameters for the ACLT group and the OARSI scores.

LCL	OARSI											
	LT		MT		LF		MF		GR		PAT	
	*r*	*p*	*r*	*p*	*r*	*p*	*r*	*p*	*r*	*p*	*r*	*p*
E_nf_ (MPa)	0.112	1.000	−0.791	0.133	**−0.900**	**0.048**	−0.410	0.500	0.000	1.000	0.564	0.367
E_1_ (MPa)	−0.671	0.300	0.527	0.467	0.700	0.233	0.667	0.267	−0.316	0.600	0.154	0.833
E_2_ (MPa)	−0.671	0.300	0.738	0.200	0.900	0.083	0.821	0.133	0.053	0.933	−0.154	0.833
E_t_ (MPa)	−0.335	0.600	0.369	0.533	0.600	0.350	0.410	0.500	−0.527	0.467	0.154	0.833
η_1_ (MPa*s)	0.112	1.000	0.791	0.133	0.600	0.350	0.103	0.900	0.527	0.467	**−0.975**	**0.033**
η_2_ (MPa*s)	0.112	1.000	−0.369	0.533	−0.500	0.450	−0.103	0.900	0.738	0.200	−0.051	1.000

MCL	OARSI											
	LT		MT		LF		MF		GR		PAT	
	*r*	*p*	*r*	*p*	*r*	*p*	*r*	*p*	*r*	*p*	*r*	*p*
E_nf_ (MPa)	−0.334	0.583	−0.494	0.300	−0.464	0.372	−0.116	0.839	−0.412	0.444	0.880	0.053
E_1_ (MPa)	0.820	0.067	−0.309	0.567	0.029	0.983	−0.696	0.139	−0.177	0.711	−0.152	0.817
E_2_ (MPa)	−0.213	0.733	0.463	0.367	0.812	0.072	0.551	0.272	0.736	0.122	−0.455	0.383
E_t_ (MPa)	0.273	0.617	0.432	0.400	0.812	0.072	0.174	0.756	0.177	0.711	−0.516	0.350
η_1_ (MPa*s)	0.820	0.067	−0.154	0.767	0.145	0.778	−0.551	0.272	0.029	0.978	−0.395	0.467
η_2_ (MPa*s)	0.334	0.583	−0.617	0.233	**−0.841**	**0.044**	−0.638	0.200	−0.059	0.922	0.213	0.733

Statistically significant correlations are presented in bold font

*LT* Lateral tibia, *MT* Medial tibia, *LF* Lateral femur, *MF* Medial femur, *GR* Groove, *PAT* Patella)

**Table 3:** Spearman’s correlations between optimized fibril-reinforced poroviscoelastic material parameters for the C-L group and the OARSI scores

LCL	OARSI											
	LT		MT		LF		MF		GR		PAT	
	*r*	*p*	*r*	*p*	*r*	*p*	*r*	*p*	*r*	*p*	*r*	*p*
E_nf_ (MPa)	−0.455	0.383	0.395	0.450	−0.647	0.189	0.370	0.467	0.577	0.250	−0.698	0.150
E_1_ (MPa)	−0.152	0.817	−0.030	1.000	−0.235	0.656	0.802	0.067	0.030	1.000	0.334	0.583
E_2_ (MPa)	0.213	0.733	0.030	1.000	0.147	0.778	−0.463	0.433	−0.698	0.167	0.030	1.000
E_t_ (MPa)	−0.334	0.583	0.334	0.533	0.441	0.411	0.494	0.367	−0.395	0.450	0.334	0.583
η_1_ (MPa*s)	−0.152	0.817	−0.152	0.833	−0.235	0.656	0.802	0.067	−0.213	0.733	0.213	0.733
η_2_ (MPa*s)	0.516	0.350	−0.577	0.250	−0.794	0.067	−0.309	0.567	0.091	0.883	−0.213	0.733

MCL	OARSI											
	LT		MT		LF		MF		GR		PAT	
	*r*	*p*	*r*	*p*	*r*	*p*	*r*	*p*	*r*	*p*	*r*	*p*
E_nf_ (MPa)	−0.152	0.817	0.152	0.833	−0.353	0.489	0.494	0.367	0.880	0.052	0.152	0.817
E_1_ (MPa)	0.273	0.617	0.152	0.833	−0.059	0.922	−0.185	0.733	0.334	0.533	0.516	0.350
E_2_ (MPa)	−0.759	0.083	0.759	0.117	−0.294	0.567	0.494	0.367	0.516	0.333	−0.698	0.150
E_t_ (MPa)	−0.030	1.000	−0.030	1.000	−0.441	0.411	0.679	0.133	0.152	0.833	0.334	0.583
η_1_ (MPa*s)	0.395	0.467	0.030	1.000	−0.265	0.622	−0.309	0.567	0.213	0.733	0.395	0.467
η_2_ (MPa*s)	−0.638	0.183	0.516	0.333	0.029	0.978	0.062	0.933	−0.334	0.533	−0.694	0.052

No statistically significant correlations were obtained between groups. (LT: Lateral tibia; MT: Medial tibia; LF: Lateral femur; MF: Medial femur; GR: Groove; PAT: Patella)

**Table 4:** Spearman’s correlations between optimized fibril-reinforced poroviscoelastic material parameters for the CNTRL group and the OARSI scores

LCL	OARSI											
	LT		MT		LF		MF		GR		PAT	
	*r*	*p*	*r*	*p*	*r*	*p*	*r*	*p*	*r*	*p*	*r*	*p*
E_nf_ (MPa)	−0.051	0.919	0.345	0.410	−0.135	0.758	−0.185	0.658	−0.412	0.333	0.391	0.336
E_1_ (MPa)	0.000	1.000	−0.153	0.721	0.246	0.553	−0.507	0.204	−0.151	0.714	−0.326	0.421
E_2_ (MPa)	0.437	0.281	0.319	0.452	−0.098	0.822	−0.099	0.824	−0.151	0.714	0.143	0.743
E_t_ (MPa)	0.129	0.771	−0.383	0.348	0.074	0.865	−0.296	0.470	0.014	0.988	−0.222	0.586
η_1_ (MPa*s)	−0.643	0.095	−0.243	0.569	0.049	0.920	−0.445	0.273	−0.206	0.631	−0.717	0.064
η_2_ (MPa*s)	−0.154	0.724	−0.064	0.890	−0.417	0.299	0.556	0.161	0.619	0.095	−0.326	0.421

MCL	OARSI											
	LT		MT		LF		MF		GR		PAT	
	*r*	*p*	*r*	*p*	*r*	*p*	*r*	*p*	*r*	*p*	*r*	*p*
E_nf_ (MPa)	0.334	0.424	0.115	0.805	−0.614	0.115	0.531	0.188	0.412	0.333	−0.156	0.714
E_1_ (MPa)	0.077	0.867	−0.128	0.781	0.577	0.142	−0.717	0.056	−0.536	0.167	0.261	0.543
E_2_ (MPa)	−0.180	0.686	0.370	0.364	−0.393	0.328	−0.099	0.824	−0.591	0.131	0.456	0.243
E_t_ (MPa)	−0.206	0.643	0.115	0.805	0.368	0.358	−0.469	0.250	−0.687	0.060	0.339	0.400
η_1_ (MPa*s)	−0.411	0.319	−0.434	0.293	0.417	0.299	−0.334	0.425	0.137	0.738	−0.326	0.421
η_2_ (MPa*s)	0.206	0.643	−0.192	0.645	0.110	0.799	0.284	0.495	0.687	0.060	−0.339	0.400

No statistically significant correlations were obtained between groups

*LT* Lateral tibia, *MT* Medial tibia, *LF* Lateral femur, *MF* Medial femur, *GR* Groove, *PAT* Patella

## Discussion

In the current study, we determined the biomechanical adaptations of the collateral ligaments in the rabbit knees at eight weeks after ACLT surgery using a fibril-reinforced poroviscoelastic material model. We also investigated changes in the cross-sectional area of the collateral ligaments following ACLT. We discovered that collateral ligaments in the ACL-transected knees increased in their cross-sectional area, the LCL exhibited less nonlinear elastic behavior, and the magnitude of the fast relaxation phase (peak-to-equilibrium stress) decreased in the MCL. These results suggest that the altered loading environment of the rabbit knee joint following ACLT causes mechanical adaptations in the collateral ligaments, but these adaptations are different for the LCL and MCL. Furthermore, significant correlations were observed between certain fibril-reinforced properties of the collateral ligaments and the OARSI scores for articular cartilage in ACL-transected knees.

Our fibril-reinforced poroviscoelastic material model was developed and validated previously for the characterization of the mechanical response of bovine knee ligaments and the patellar tendon.^43^ This same material model captured well the sample-specific tensile stress-relaxation response of collateral ligaments in ACL-transected, contralateral, and healthy rabbit knees, with distinct fast and long-term relaxations and high R^2^-values. Our results support that this constitutive model can be applied to evaluate the progressive changes in the biomechanical properties of ligaments associated with the adaptative processes following a traumatic joint injury.

The MCL and LCL in the ACL-transected knees had larger cross-sectional areas compared with the corresponding ligaments obtained from the contralateral and control group knees. Specifically, values for the cross-sectional area of the MCL were 1.9 and 1.6 times greater compared to the contralateral and control group values, respectively. This finding is in good agreement with previous observations reported by Bray *et al.*^5^, where the cross-sectional area of the MCLs were 1.5 times greater than controls six to 14 weeks after ACLT, and almost 2 times greater than controls from 14 to 48 weeks after ACLT in rabbits. In agreement with our results, Bray *et al.*^5^ also reported negligible decrease in stiffness of MCL in ACLT joints during 6–48 weeks after surgery. In addition, values for the cross-sectional area of the LCL were 2.5 and 1.9 times greater than those of the contralateral and control group values, respectively. This result is also consistent with a previous investigation where the cross-sectional areas of the LCLs were 1.4 times greater compared to those of sham-operated control specimens, eight to 12 weeks after ACL and posterior cruciate ligament (PCL) transections in rabbits.^56^ However, the same study reported equal LCL cross-sectional area at 16 weeks to control values. In addition, in agreement with our findings, Atarod *et al.*^2^ described significant changes in sheep LCL load magnitudes during gait at 20 weeks following ACLT but MCL load magnitudes remained unaltered during the same time frame after ACLT during gait. It can be hypothesized that following transection of the ACL, there is a fast physiological adaptation of the collateral ligaments caused by alterations in the varus-valgus angle, posterolateral rotations, as well as in the loading conditions of the knee joint. Although both collateral ligaments had larger cross-sectional areas following ACLT, the LCL became weaker per unit of area and its stiffness decreased after ACLT. The stiffness of the MCL remained unaltered following ACLT probably due to the knee joint attempting to maintain normal function by adapting either the modulus or the cross-sectional area. However, it is worth mentioning that further changes may also occur in intact ligaments for longer periods of time after ACLT. For instance, Tapper *et al.*^54^ reported changes in the distance between insertions of the remaining intact ovine LCL and PCL at 20 weeks after ACL/MCL transection. In addition, the MCL and LCL cross-sectional areas in the contralateral group were lower than in the control group, although, at the time of evaluation, the difference was not statistically significant. This result may indicate that after ACLT, alterations in the locomotion affects both knees and causes different adaptations in the collateral ligaments of the experimental (ACLT) and the contralateral knee. However, this finding might also result from normal variation between animals. To fully clarify this, additional experiments would be necessary.

The LCL in the ACL-transected knees had a decrease in the elastic behavior (${E}_{\mathrm{t}}$), as expected based on a previous investigation where the LCL was softer in the unstable ACLT knees compared to the sham-operated knees at eight weeks after transection.^56^ This softening could be caused by a decrease in the elastin content of the LCL, as elastin contributes to the elastic behavior.^14,46^ Conversely, the elastic response of the MCL was similar between the groups. This finding is consistent with a previous study where a decrease in MCL stiffness was negligible at six to 48 weeks after ACLT surgery.^5^ Nevertheless, structural changes in the waviness of the collagen network, measured via polarized light microscopy, as well as variations in the composition (collagen, proteoglycan, and elastin contents) would be useful measures to complement our understanding of the adaptative processes of the mechanical properties in the collateral ligaments. Such insight would provide additional information on the multi-scale physiological adaptations of the collateral ligaments in ACL-deficient knees.

The MCL specimens in the ACLT group showed a significant decrease in the magnitude of the fast relaxation phase (${E}_{1}$) but the damping coefficient ${\eta }_{1}$ was similar between the groups. This finding agrees with the early variations in the viscoelastic properties of MCL in ACL-transected rabbit knees six weeks after surgery.^5^ We speculate that these changes in the fast relaxation properties are related to alterations in the collagen fibrillar network organization and the inter-fibre sliding within the fibrillar network due to increased tensile loads on the MCL.^43,49^ Conversely, the amount of long-term relaxation (${E}_{2}$) and the long-term damping coefficient ${\eta }_{2}$ of both collateral ligaments was similar in all experimental groups. This result indicates that the long-term relaxation properties of collateral ligaments remain unaltered or change slowly, possibly originating from a gradual structural reorganization of the collagen network following ACLT.

Ligament fibril-reinforced poroviscoelastic material parameters were associated with OARSI scores in ACLT group animals. The significant negative correlations between the non-fibrillar matrix modulus and the damping coefficient for fast relaxation of the fibrillar network of the LCL with the OARSI scores of the lateral femoral condyle cartilage and patella, respectively, may indicate that following ACLT, the LCL become softer during dynamic/fast loading and it might increase varus angulation and internal tibial rotation which may accelerate cartilage degeneration. However, we acknowledge that our findings are indirect and not conclusive. Therefore, further investigation is needed to determine the complex interplay that exists between ligament remodeling properties and the mechanisms for osteoarthritis progression. Experiments with additional samples and time points after ACLT surgery could help elucidate these complex interactions.

Viewed together, our results provide insights into the adaptation of mechanical properties of ligaments after traumatic injuries using numerical simulations. This approach could be implemented in computational knee joint models to evaluate the effect of compositional alterations in ligament properties and associated ligament function on the global knee joint mechanics and the subsequent impact on other knee joint tissues, such as the articular cartilage, bone, or menisci.^4,38^ Furthermore, our results are relevant when investigating the mechanobiological response in human knee joints to estimate adaptation, damage, or degeneration of knee joint tissues, evaluating different timescales after injury or disease. For example, we expect that structural and compositional alterations in cartilage, menisci, and bone might occur earlier compared to changes in ligaments and tendons.^4,18,25^ However, we acknowledge that this investigation was conducted using an animal model and therefore our findings may not be directly translatable to the human knee.

There were also some limitations in this study. First and foremost, we did not determine nor consider alterations in the composition and structure of the collateral ligaments in our numerical simulations. For instance, significant changes in the water content of the MCL in ACL-deficient knees have been reported.^33^ Alterations in the biochemical composition and structure, for example, water, collagen, elastin, and proteoglycan contents and crimp characteristics following ACLT will be characterized in the future to better explain variations in the material model parameters.^43^ Also, our FE models used simplified elliptical geometries similarly to those used in previous studies.^3,21,22,36,45,55^ More realistic ligament geometries could be generated from microCT. Nevertheless, sample-specific lengths and cross-sectional areas were considered in the FE geometries for all the ligaments analyzed in this study and the conclusion about the adaptation of viscoelastic properties should not be affected by this limitation. Moreover, there were limitations in the experimental methods and specific assumptions in the numerical models that must be considered when interpreting our results. First, rabbit ligaments are not equivalent to human tissue. The anatomy and size of the rabbit knee, as well as the locomotion patterns, differ between rabbit and human knees.^41^ These differences must be acknowledged when applying our findings to the human knee. However, ligaments, tendons, basic bones, and muscular structures are similar in these two species.^7,11,41^ Hence, the physiological variations described here could have importance in the adaptation of collateral ligaments of the human knee after an ACL injury. Second, the non-fibrillar matrix modulus described only a small effect on the overall mechanical response, which cannot be uniquely optimized in the material model. This may result in large variations when determined using optimization procedures, and therefore actual differences between ligaments may have been obscured. Third, the small number of animals and the short time frame after ACLT surgery represent limitations in our study. The structural adaptation of collateral ligaments is limited to the intrinsic variability among rabbits and a single time point after the surgery. Regarding sample size, if the data from our study were to be used as pilot information for future research, a power analysis would be useful. With the current sample size of 6 transected and 4 control animals, there is a 96% power to detect a difference in cross-sectional area between the groups. In contrast, analysis for the change in stiffness shows 10% power. The sample size required to obtain a power of 90% for detecting changes in stiffness would be 380. Thus, there is likely no difference in stiffness between the groups. Therefore, our results should be interpreted with caution because of these limitations. More samples and later time points post-ACLT are required to investigate variability between subjects and changes as a function of time. Finally, previous studies suggested that invasive ACLT may introduce confounding factors compared to less invasive mechanical ACL rupture.^30^ In our investigation, potential surgery-induced alterations such as pain, inflammation, and unloading were not considered due to the lack of a sham operated control group. However, we believe that these potential effects are minor and would not have affected the conclusions of our study. Previous evidence^5,56^ suggests that there are no significant differences in the mechanical properties of rabbit MCL and LCL from normal controls and sham-operated rabbits for 6–48 weeks^5^ and 4–16 weeks^56^ after ACLT surgery, respectively. Nevertheless, future studies should include a sham surgery group in the experimental design to consider potential surgery-induced changes on the results.

In conclusion, our results indicate that potential alterations in the motion and loading environment of the knee joint after an ACL injury cause early adaptations of the elastic and viscoelastic properties of the collateral ligaments, but the adaptations differ between the LCL and MCL. Our findings improve the understanding of functional alterations in collateral ligaments after ACL injury. These alterations could have an impact on cartilage degeneration and the progression of osteoarthritis, and thus, should be monitored using clinical imaging techniques. The impact of collateral ligament adaptations on the whole knee joint function could be evaluated using computational modeling to reveal candidate mechanisms associated with osteoarthritis progression and its prevention or acceleration.
